# Introduction to the Special Issue “Nutritional Status and Eating Patterns in Children and Adolescents: Prevalence, Screening and Prevention of Metabolic Disorders”

**DOI:** 10.3390/children12050539

**Published:** 2025-04-23

**Authors:** Stefania Moramarco, Ersilia Buonomo

**Affiliations:** Department of Biomedicine and Prevention, University of Rome Tor Vergata, 00133 Rome, Italy

Nutrition plays a fundamental role in shaping the current and future health of children and adolescents. A sustainable, balanced diet and healthy eating behaviors are essential not only for preventing nutritional deficits and linear growth faltering early in life, but also for mitigating the risk of chronic noncommunicable diseases in adulthood.

This Special Issue of *Children* presents a diverse collection of articles that examine the nutritional status and eating patterns of children and adolescents from multiple perspectives, highlighting the complexity and urgency of the issue. It emphasizes the need for a holistic approach that bridges nutrition-specific strategies with broader public health, education, and clinical frameworks, offering a comprehensive approach to tackle nutrition-related challenges from multiple angles.

Childhood obesity represents one of the most prevalent and complex chronic diseases, associated with serious short- and long-term morbidities, as well as an increased risk of early mortality. It is a well-recognized major growing public health concern in Western countries. In the systematic review of Saltaouras et al. [1], the associations between meal patterns and the risk of childhood overweight/obesity (OV/OB) were assessed in children/adolescents. The findings identified a protective effect of shared family meals, while the results regarding meal frequency and timing were inconclusive. Notably, the review highlighted gender differences, particularly in breakfast skipping, suggesting that girls may be more influenced by societal dieting pressures. A gender gap was also found by Cakoni et al. [2] in a cross-sectional study assessing eating habits and nutritional status among Albanian youths, a country undergoing nutrition transition. The authors reported an increase rate of OV/OB with a suboptimal adherence to the principles of the Mediterranean Diet, with worse results for girls, especially when living in rural areas. These results highlight the need to prioritize healthy nutrition lifestyles as a cornerstone for ensuring the long-term wellbeing of younger generations, with nutritional interventions and policies taking specifically into account geographic, cultural, and social factors—including gender equality—that can influence dietary patterns. By specifically focusing on female gender and nutrition, Guembri et al. [3] assessed changes in sleep quality and physical performance in female handball players fasting during Ramadan. The results showed a decrease in sleep quality over the weeks, highlighting the importance of maintaining low-intensity training and ensuring adequate recovery to preserve athletic performance in adolescent girls during fasting periods. Complementing this, Remmel et al. [4] investigated disordered eating patterns and hormonal profiles in adolescent rhythmic gymnasts. Compared to untrained peers, gymnasts exhibited more frequent restrained eating patterns, also associated with a higher serum irisin concentration.

Obesity demonstrates significant heterogenicity, and youths classified as having metabolically healthy obesity (MHO) exhibit a more favorable metabolic profile than those with metabolically unhealthy obesity (MUO), which is defined as the absence of components of the metabolic syndrome. However, as shown in the article of Bartogianni et al. [5], even the MHO group exhibits metabolic alterations, highlight that all children with obesity—regardless of the most recent classifications—require close monitoring for both short- and long-term complications. Contributing to the increasing trend of OV/OB is the increased consumption of sugar-sweetened beverages. In a prospective study, Hermansen et al. [6] assessed the relationship between soft drink intake and bone health in Danish schoolchildren. Their findings revealed no significant impact on bone mineral content or bone mineral density, nor an increased fracture risk. However, the authors advocate for future research to differentiate between carbonated and non-carbonated beverages and their potential long-term effects.

The shift in dietary patterns and increasing OV/OB rates are not limited to high-income countries. In their cross-sectional study, Morais et al. [7] analyzed data from a sample of children and adolescents attending public schools in São Tomé and Príncipe. They found a high consumption of ultra-processed foods, which was associated with a lower intake of fiber, vitamin B12, and zinc, and a higher intake of iron and sodium, raising the risk of some metabolic and nutritional diseases and reflecting the coexistence of a double burden of malnutrition in new generations in Sub-Saharan Africa. In the specific context of Sub-Saharan Africa and undernutrition, the study conducted by Negesse et al. [8] in Ethiopia examined the potential implications of the new WHO 2023 guidelines for the management of infants under six months with malnutrition. The WHO-2023 criteria markedly increase malnutrition caseloads compared with the WHO-2013 criteria. However, the study underscores the need for local epidemiological assessments and robust planning before the large-scale implementation of new clinical protocols, as there may be a case for increasing MUAC thresholds in MUAC-focused programs where the measurement of WAZ and WLZ is sometimes difficult. The authors emphasize that future longitudinal data are needed to determine which criteria best identify infants at the highest risk of mortality, morbidity, and poor development.

Additionally, this Special Issue promotes a vision of holistic, inter-professional care, where nutrition is not only a standalone concern but a core component of broader health and developmental strategies. In their scoping review, Hung et al. [9] explored the relationship between nutritional deficiencies and adolescent oral health. They found strong associations between malnutrition and dental caries, calling for early and integrated interventions that connect dietary guidance with dental care. Lastly, Pergeline et al. [10] offer a comprehensive narrative review of pediatric feeding disorders (PFDs), tracing the evolution of their classifications. Despite the availability of general feeding guidelines, PFDs remain under-recognized, and the authors call for increased awareness and specific clinical protocols. Their review promotes a transdisciplinary model of care, integrating medical, nutritional, feeding skills, and psychosocial assessments to foster a unified approach to diagnosis and treatment.

This collection of research will be of value to researchers, educators, clinicians, and policymakers seeking to improve nutritional health outcomes across diverse populations and contexts. It also serves as a call to action for designing comprehensive, evidence-based interventions that are gender-sensitive, culturally tailored, and sustainable over the life course.

## Data Availability

Not applicable.

## References

[1] Saltaouras G., Kyrkili A., Bathrellou E., Georgoulis M., Yannakoulia M., Bountziouka V., Smrke U., Dimitrakopoulos G., Kontogianni M.D. (2024). Associations between Meal Patterns and Risk of Overweight/Obesity in Children and Adolescents in Western Countries: A Systematic Review of Longitudinal Studies and Randomised Controlled Trials. Children.

[2] Cakoni R., Moramarco S., Kosiqi A., Andreoli A., Buonomo E. (2025). Dietary Habits and Nutritional Status of Youths Living in Rural and Semi-Urban Albania in the Ongoing Nutrition Transition: Preliminary Results. Children.

[3] Guembri M.A., Racil G., Tounsi M., Aouichaoui C., Russo L., Migliaccio G.M., Trabelsi Y., Souissi N., Padulo J. (2024). Effects of Ramadan Fasting on Sleep and Physical Fitness among Young Female Handball Players. Children.

[4] Remmel L., Jürimäe J., Tamm A.-L., Purge P., Tillmann V. (2024). High Serum Irisin Concentration Is Associated with More Disturbed Behavioural Eating Pattern in Adolescent Rhythmic Gymnasts. Children.

[5] Baltogianni M., Dermitzaki N., Giapros V., Balomenou F., Kosmeri C., Ladomenou F., Kantza E., Serbis A. (2025). Indicators of Glucose Metabolism in Children and Adolescents Characterized as Having “Metabolically Healthy” and “Metabolically Unhealthy” Obesity. Children.

[6] Hermansen H., Händel M.N., Heidemann M.S., Wedderkopp N. (2025). Impact of Soft Drink Intake on Bone Development and Risk of Fractures in a Danish Cohort of Schoolchildren. Children.

[7] Morais R., Rodrigues M., Ferreira F., Barros R., Padrão P., Ortigão M., Tavares M., Moreira P. (2024). Ultra-Processed Foods and Nutritional Intake of Children and Adolescents from Cantagalo, São Tomé and Príncipe. Children.

[8] Negesse A., Girma T., Desalegn B.B., Berhane M., Kerac M. (2025). The Impact of WHO-2023 Malnutrition Criteria on Caseload of Infants Aged Under Six Months: Secondary Data Analysis. Children.

[9] Hung M., Blazejewski A., Lee S., Lu J., Soto A., Schwartz C., Mohajeri A. (2024). Nutritional Deficiencies and Associated Oral Health in Adolescents: A Comprehensive Scoping Review. Children.

[10] Pergeline H., Gonnet L., Fernandez A., Solla F., Poinso F., Guivarch J. (2025). Diagnosis and Treatment of Pediatric Feeding Disorders: A Narrative Literature Review. Children.

